# Premature baby with extreme hyponatraemia (95 mmol per litre): a case report

**DOI:** 10.1186/s12887-015-0437-1

**Published:** 2015-09-16

**Authors:** Arthur Abelian, Cristian Eugen Ghinescu

**Affiliations:** Department of Paediatrics, Wrexham Maelor Hospital, Betsi Cadwaladr University Local Health Board, Croesnewydd Rd, Wrexham, LL13 7TD UK; Alder Hey Children’s Hospital NHS Foundation Trust, Eaton Rd, Liverpool, L12 2AP UK

**Keywords:** Hyponatraemia, Hypovolaemia, Urinary tract infection, Donor breast milk, Prematurity

## Abstract

**Background:**

Whilst mild neonatal hyponatraemia is common and relatively harmless, extreme hyponatraemia of 95 mmol per litre has never been reported in a premature baby and such a level could be associated with immediate as well as long-lasting detrimental effects on health.

**Case presentation:**

Twenty-four days old baby boy born at 28 weeks gestation (triplet one) unexpectedly became moribund with hypovolaemic shock and was found to have blood sodium of 95 mmol per litre. Diagnostic work up revealed a combination of a urinary tract infection, inadvertently low sodium provision with donor breast milk, and weak renin-angiotensin-aldosterone response. Commencement of treatment with intravenous fluids and extra sodium led to unanticipated diuresis and faster than expected increase of sodium level. Ultimately, treatment resulted in clinical recovery and normalisation of sodium level, which subsequently remained normal with no additional sodium supplementation. Follow up revealed mild spastic diplegia.

**Conclusion:**

Continuous monitoring and daily medical reviews may not be sensitive enough to recognise development of extreme hyponatraemia. Blood sodium levels should be monitored closely and any abnormalities promptly addressed. Treatment of hypovolaemic hyponatraemia should be centred on fluid resuscitation, anticipation of “paradoxical” diuresis, and blood sodium correction rate of 8 to 10 mmol per litre per day.

## Background

Mild hyponatraemia is common in preterm babies and is not known to cause significant adverse effects [1]. On the contrary, extreme hyponatraemia is rarely seen and increases the risk of neurodisability [2, 3]. Many factors predispose preterm babies to hyponatraemia: impaired reabsorption of sodium at both proximal and distal tubules [4], inadequate salt provision, *e.g.* with donor breast milk (DBM) [5], immaturity of endocrine mechanisms of water and sodium homeostasis [1, 6].

Reported here is a case of a DBM-fed preterm baby who developed hypovolaemic shock and extreme hyponatraemia of 95 mmol per litre – such a level of hyponatraemia has not been reported in a premature baby before and posed significant diagnostic and management challenges.

## Case presentation

### Background

Baby boy, birth weight 1257 g (close to 75^th^ centile), triplet one, delivered at 28 weeks gestation by emergency caesarean section for antepartum haemorrhage, in good condition at birth. On day nine he was weaned off the ventilator onto nasal continuous positive airway pressure support (CPAP) and then fully weaned off CPAP by day 23 of life. Except for episodes of bradycardia on day three of life, which did not require cardio-pulmonary resuscitation, and slow weight gain, his progress had been fairly unremarkable. At the time of presentation he was 24 days old (corrected gestational age 32 weeks), weight was 1360 g (close to 9^th^ centile) and he was still nursed in the incubator.

### Presentation

On the morning ward round the baby was noted to be irritable with high-pitched cry, pale-mottled skin, and mild skin tenting on the abdomen. Pulse oximetry readings were 100 % in room air, respiratory rate was 30 – 45 breaths per minute, heart rate 140 – 145 beats per minute, all peripheral pulses were palpable, accurate blood pressure measurement could not be obtained, there was a formed stool in the nappy. Serum sodium was 95 mmol per litre (using indirect ion-specific electrodes [ISE] method), which was corroborated on the point-of-care analyser (using direct ISE method; Roche OMNI S, Roche Diagnostics Ltd.): 94.5 mmol per litre. The latter specimen of blood, when tested in the hospital laboratory, returned a sodium of 95 mmol per litre with no interference from lipids, blood sugar or proteins; osmolality of the same specimen was measured using freezing point depression osmometer and was found to be very low - 203 milli-osmoles per kg - consistent with hypotonic hyponatraemia and against a factitious cause.

### Diagnostic work-up

**Fig. 1:**
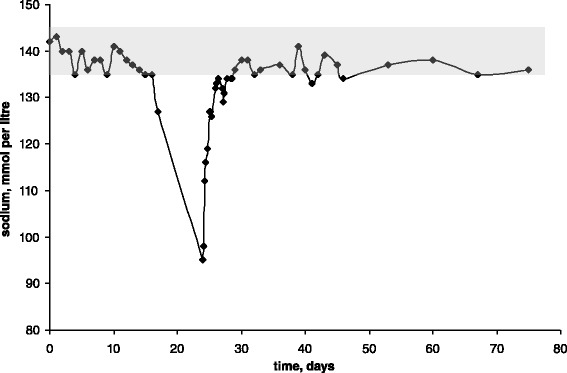
Time line of sodium concentration in the blood. Shaded area represents the normal range (135 – 145 mmol per litre)

Table 1 presents standard laboratory data obtained on presentation, and then 12, 24 and 96 h after treatment initiation. Figure 1 shows a timeline of blood sodium levels. From day one of life and until this baby was fully enterally fed, serum sodium was measured daily (*i.e.* until day 17). Sodium level remained within the normal range except for day 17 when it was 127 mmol per litre. No more measurements were done until day 24 and it was therefore not possible to describe the rate of sodium drop accurately.

**Table 1:** Laboratory data

Variable	Reference range	On presentation	12 h treatment	24 h treatment	4 days treatment
Na (mmol/litre)	135 – 145	94	116	128	134
K (mmol/litre)	3.5 – 5.3	5.2	3.6	4.7	2.8
Creatinine (micromole/litre)	58 – 110	16	24	20	23
Urea (mmol/litre)	2.5 – 7.8	3	1.9	1.5	1.4
Serum osmolality (mosmole/kg)	275 – 295	203		258	
Blood glucose (mmol/litre)	3.0 – 7.7	3.9	6.1		4.5
Albumin (g/litre)	35 – 50	34	26		21
Calcium, adjusted (mmol/litre)	2.1 – 2.65	2.55			2.55
Phosphate (mmol/litre)	0.8 – 1.5	1.45			2.46
Chloride (mmol/litre)	95 – 108	68	88		108
pH	7.35 – 7.45	7.36	7.28	7.29	7.35
pCO_2_ (kPa)	4.5 – 6.1	4.1	5.7	6.1	6.2
Bicarbonate (mmol/litre)	24 – 32	16.8	19.5	21.9	25.3
Base excess (mmol/litre)	−2.0 – 2.0	−7.8	−8.2	−5.9	−1.6
Lactate (mmol/litre)	0.4 – 2.2	4.4			1.3
Haemoglobin (g/dl)	11.5 – 16.5	15			
White cell count (x10^9^/litre)	5.0 – 18.0	21.3			
Neutrophils (x10^9^/litre)	1.5 – 10	10.9			
Lymphocytes (x10^9^/litre)	3.0 – 10.0	9.8			
Platelets (x10^9^/litre)	150 – 500	262			
C-reactive protein (mg/litre)	0.0 – 10.0	2.2	5.5	20.5	3.2
17-OH progesterone (nmol/litre)	0.0 – 20.0	20.9			
Cortisol (nmol/litre)	not applicable		514		
Aldosterone (nmol/litre)	0.083 – 0.44				1.15
Renin (nmol/litre/h)	0.5 – 4.4				>28.8
TSH (μU/ml)	0.35 – 5.5		2.4		
Free T3 (pmol/litre)	3.5 – 6.5		3.2		
Free thyroxine (pmol/litre)	7.0 – 17.0		11.5

**Fig. 2:**
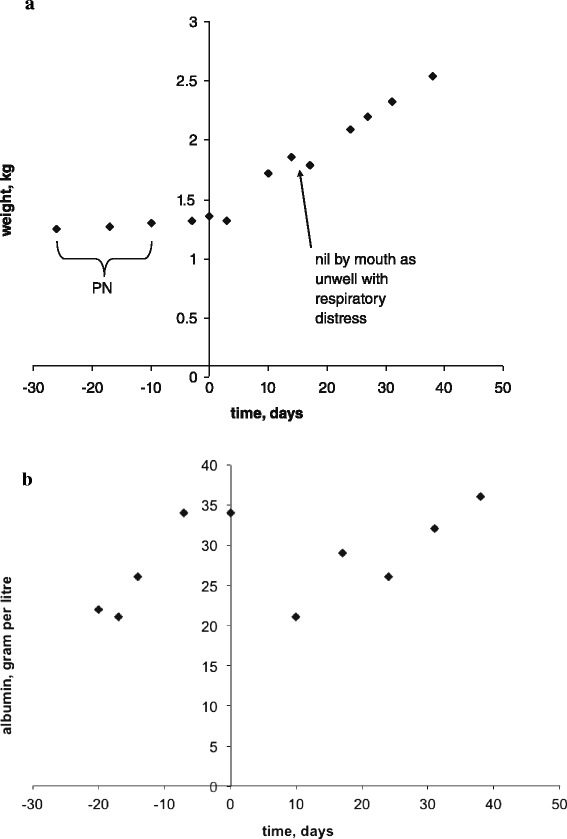
Nutritional status. Day 0 stands for day of presentation. **a** – weight gain/loss over time; PN stands for parenteral nutrition. **b** – serum albumin change over time
  On presentation, a transurethral catheter was passed and only a very small volume of urine could be obtained. However, on commencement of treatment with intravenous 0.9 % sodium chloride (see below), very brisk urine output was noted averaging 25 ml per hour in the first five hours. Based on the available data, the fluid balance over the first 27 h after presentation and commencement of treatment was a net loss of 87 ml (Fig. 3). Brisk diuresis has been described in patients with hypovolaemic hyponatraemia shortly after the commencement of treatment, as the expansion of the circulatory volume improves perfusion, thus inhibiting ADH and allowing normal osmoreceptor response to low plasma sodium [9–11].

Blood sodium levels depend on sodium and water intake and excretion [1]. The excretion is mainly regulated by antidiuretic hormone (ADH) and renin-angiotensin-aldosterone system and is geared towards maintenance of circulatory volume [7]. The intake is regulated by thirst but in an artificially fed baby would be determined by the volume and tonicity of feeds [1]. Pathogenesis of hyponatraemia in this baby has been explored:

1. *Sodium dilution due to excessive fluid intake/retention.*
  Despite receiving between 180 and 200 ml per kg per day of milk this baby had a rather poor weight gain: in 11 days before presentation his weight gain was a mere 60 g (Fig. 2a), whereas target weight gain is around 30 g per day [8]. His hydration status on presentation was described as reduced (mild abdominal skin tenting) and he had clinical features of early hypovolaemic shock with vasoconstriction: tachycardia, pallor, mottled skin and hypothermia (in the preceding 36 h the incubator temperature had to be increased from 30 to 32.5 °C). Capillary blood gas on presentation revealed compensated metabolic acidosis with raised lactate in keeping with tissue hypoperfusion (Table 1). In addition, poor weight gain notwithstanding, albumin was essentially normal on presentation consistent with intravascular volume contraction rather than nutritional sufficiency (Table 1 and Fig. 2b). In the 11 days that followed presentation, as his condition normalised and sodium deficit replenished, he gained 540 g (Fig. 2a).

**Fig. 3:**
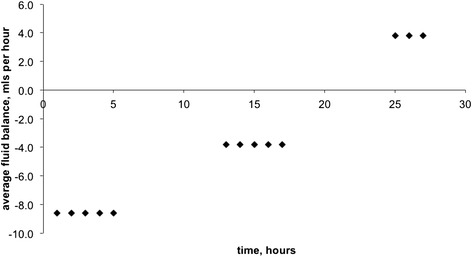
Fluid balance after presentation
  Taken together, these features argue against sodium dilution as the sole reason for this baby’s extreme hyponatraemia, and point to hypovolaemia with reduced total body sodium. The latter could have resulted from insufficient intake and/or excessive losses of sodium.

**Fig. 4:**
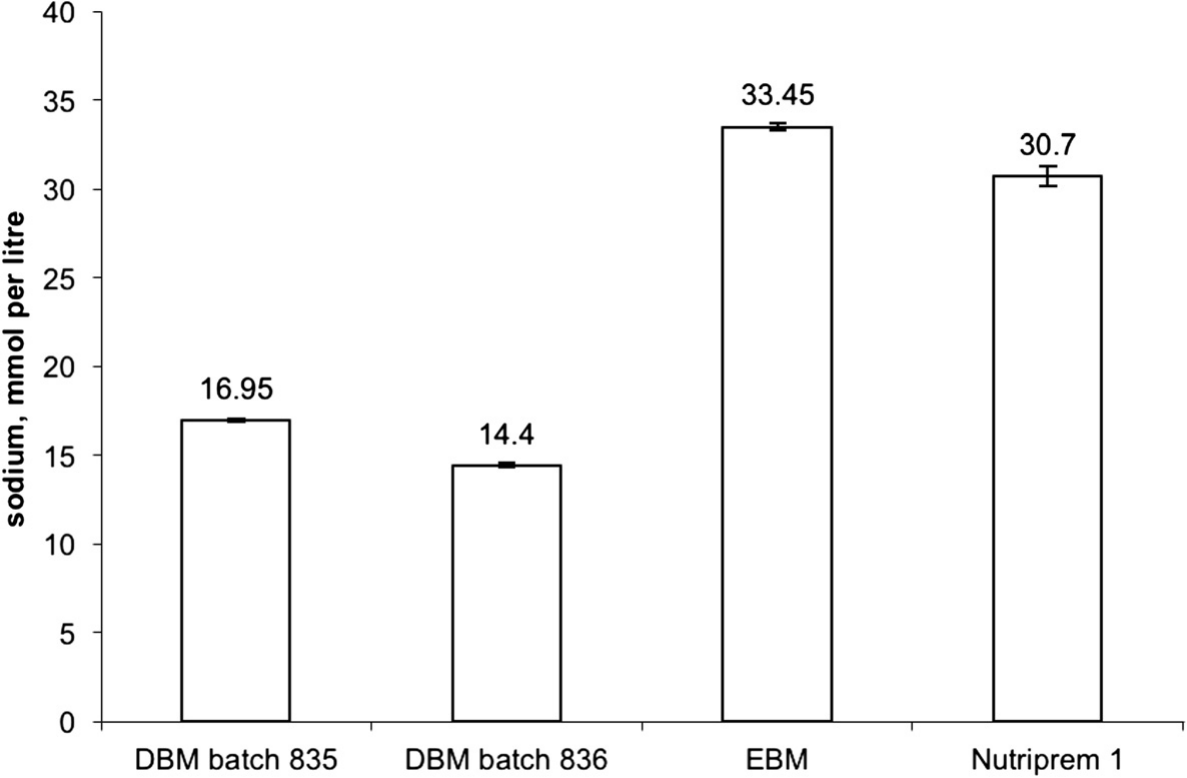
Comparison of sodium concentration in the two batches of DBM, maternal expressed breast milk (EBM) and preterm formula (Nutriprem 1). Denoted are mean values and ranges (based on two measurements). All measurements were done using Roche OMNI S point-of-care analyser

**Fig. 5:**
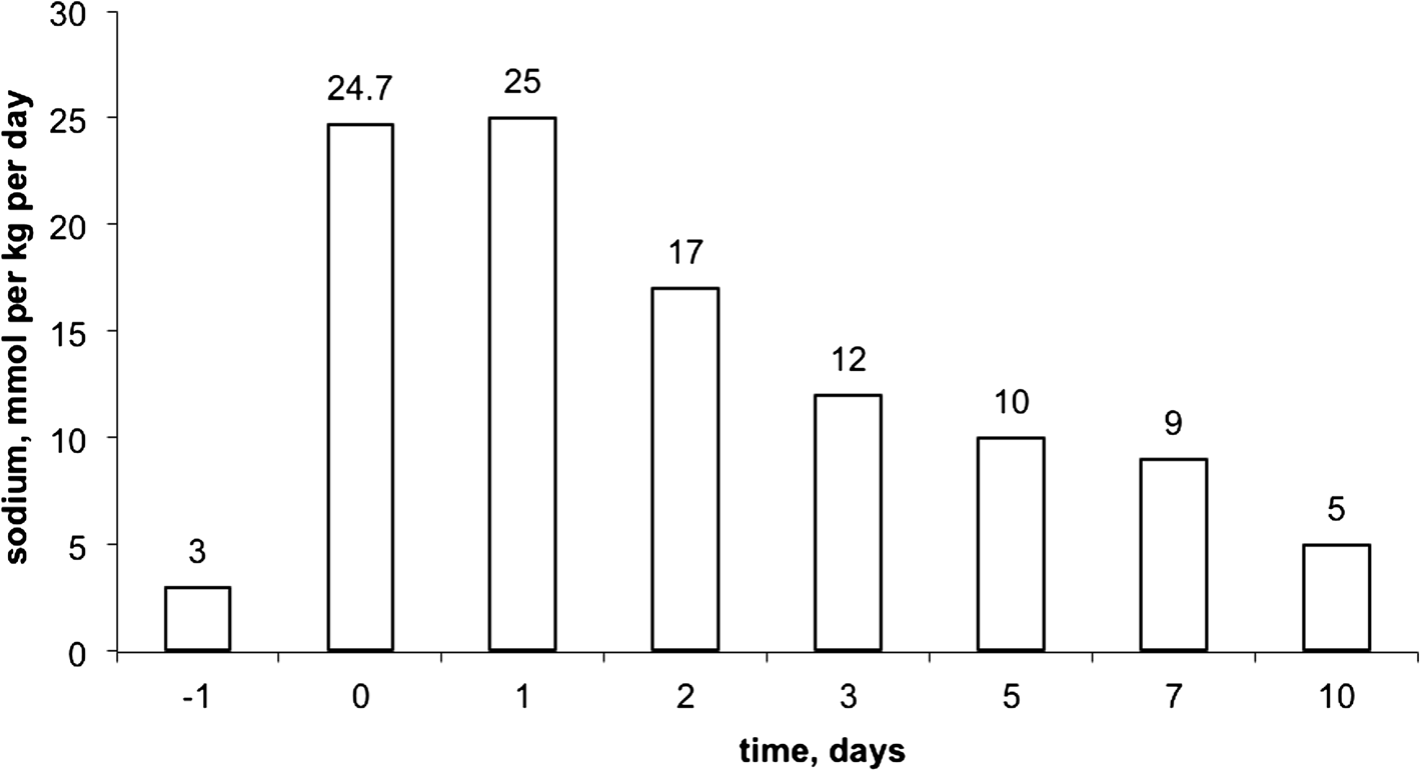
Total sodium supplementation. Day of presentation with hyponatraemia is denoted as day 0
  Known causes of sodium loss *via* kidneys have been considered below:
  - Congenital adrenal hyperplasia (CAH) could lead to the deficiency of cortisol and aldosterone presenting with hyponatraemia, metabolic acidosis, hyperkalaemia and hypoglycaemia [17]. Urine steroid profile tested at the time of hyponatraemia and 48 h later when plasma sodium normalised found no evidence for any of the inborn errors of steroid metabolism associated with CAH (Table 4). In addition, in more than 90 % of CAH, serum 17-α-OH progesterone is markedly elevated but was essentially normal in this baby and so was potassium (Table 1). Blood sugar was 3.9 mmol per litre on presentation but rose briefly to 17.9 within four hours of treatment before spontaneously normalising to 5.1 mmol per litre four hours later. These results, as well as subsequent full recovery, were decidedly against CAH.
  - Significant electrolyte disturbances have been described following the use of diuretics [7]. This baby had never received loop or thiazide diuretics. In the two weeks before presentation his only medication had been caffeine base at 2.5 mg/kg once a day orally as treatment of apnoea of prematurity. Caffeine is known to have mild diuretic and natriuretic effects [18], however to our knowledge, at the above dose, has never been described as a cause of clinically significant hyponatraemia.
  - Hyponatraemic hypertensive syndrome (HHS) [19] is caused by unilateral renal ischaemia, most commonly in neonates due to renal artery thrombosis following umbilical artery catheterisation. In this case there was no evidence of renal ischemia on renal ultrasound scan performed within 24 h of presentation (not shown) and whilst there were documented difficulties in obtaining accurate blood pressure on presentation, at 20 h of treatment blood pressure was 51/35 mmHg and baby’s condition resolved with fluids, electrolytes and antibiotics: all arguing against the diagnosis of HHS.
  - Urinary tract infection (UTI) in babies can lead to hyponatraemia due to impairment of tubular sodium reabsorption (both proximally and distally) [20]. As epithelial distal channels become transiently insensitive to aldosterone, its level goes up reciprocally (pseudohypoaldosteronism). Affected babies also develop hyperkalaemia and metabolic acidosis [20]. This baby had a number of features consistent with UTI. On presentation, blood and urine (obtained aseptically *via* transurethral catheter) were sent off for bacterial culture and the baby was treated empirically with intravenous co-amoxiclav and gentamicin. Blood culture was negative after 5 days of incubation but urine culture yielded E. coli and Enterobacter cloacae – both are recognised causes of UTI; multiple growth in properly collected urine samples could represent true mixed UTI [21]. Furthermore, coincidently with the earliest sodium drop (to 127 mmol per litre; day 17 of life) this baby developed leucocytosis (Table 3). C-reactive protein (CRP) level was 2.2 mg per litre on presentation (day 24 of life) but rose to 20.5 by 28 h before falling to 15 mg per litre at 44 h. Review of CRP and total white cell count of his triplet sisters showed normal values throughout their stay on the neonatal unit (not shown).

2) *Insufficient sodium intake.*
  At the time of the hyponatraemic crisis the baby had been fully enterally fed for a week receiving between 180 and 200 ml per kg per day of donor breast milk (DBM; derived from two batches) and maternal expressed milk or low-birth weight formula (Nutriprem 1, Cow&Gate, UK). The ratio of DBM to other milks was four to one. The reported sodium content of Nutriprem 1 is 70 mg per 100 ml [12], which converts to 30 mmol per litre.
  Figure 4 shows that the sodium content of DBM he was fed was half of that in either Nutriprem 1 or mother’s expressed breast milk (EBM). Thus for the week preceding presentation with extreme hyponatraemia he had been receiving between 2.7 and 3.3 mmol per kg of sodium per day. The daily provision of sodium should be between 4 and 6 mmol per kg per day [13] and therefore this baby’s sodium provision was short of adequate. This notwithstanding, suboptimal sodium provision could not have been the sole explanation of the extreme hyponatraemia as his triplet sisters, who were fed DBM in the same ratio with Nutriprem 1 and EBM, maintained the serum sodium between 132 and 138 mmol per litre (not shown). Indeed, in the seven days when his sodium dropped from 127 to 95 mmol per litre, to avoid the drop his total sodium intake had to be close to 82 mmol: in these seven days he received 48 mmol and accrued a deficit of 35 mmol ((127 mM – 95 mM) × 1.36 kg × 0.8 (assuming extracellular water 80 % of body weight [14]). This equates to a daily requirement of 9 mmol per kg per day and indicates that the deficit could be accounted for, roughly in equal measure, by the inadequate provision and losses.

3) *Sodium losses: where, how much and how?*
  Review of the baby’s records revealed neither vomiting nor diarrhoea, and therefore any excessive sodium loss was *via* the kidneys. Fractional excretion of sodium (FENa) has been used to measure the proportion of filtered sodium that is excreted in the urine [15]. Values of FENa up to 3 % were described in critically ill preterm babies without acute renal failure (ARF) and values 6.9 ± 2.9 % in preterm babies with ARF [16]. At 27 h after presentation FENa was 1.3 and at 47 h 2.3 %. However, these figures are difficult to interpret: during those 47 h the baby received approximately 50 mmol of sodium per kg of weight (Table 2 and Fig. 5) and FENa is positively related to sodium provision [7]. The concentration of sodium in the first urine post presentation was 19 mmol per litre and osmolality 160 milliosmoles per kg (Table 2) but it was collected during the phase of brisk diuresis that followed commencement of treatment with 0.9 % sodium chloride infusion and hence was not helpful for the analysis of sodium excretion either.

**Table 2:** Blood and urine sodium, creatinine, and osmolality, and FENa

Time, hrs	Blood			Urine			FENa, %
	Sodium, mmol/l	Creatinine, μmol/l	Osmolality, mosm/kg	Sodium, mmol/l	Creatinine, μmol/l	Osmolality, mosm/kg	
0	94	16	203/*207* ^a^	n/d^c^			
4	112	20	n/a^b^	19	<880	160	n/a
9	116	24	*238*	n/d			
23	128	20	258/*265*	n/d			
27	127	21	*265*	93	1110	418	1.3
33	126	17	*260*	<10	<880	n/a	n/a
47	133	17	*274*	178	980	465	2.3
72	131	34	261/*268*	37	<880	139	n/a
98	134	23	*274*	128	1610	440	1.3

^a^In italics - calculated osmolality

^b^n/a – not available

^c^n/d – not done

**Table 3:** C-reactive protein (CRP), white blood cells, haemoglobin and blood sodium over time

	Age (days)								
	11	13	17	24	26	37	40	46	61
Neutrophils, x10^9^/L	6	11.1	12.4	10.9	n/d^b^	n/d	n/d	4.2	1.9
WCC^a^, x10^9^/L	15	21.3	30.3	21.3	n/d	15.9	4.9	16.6	13.4
Hb, g/dL	16.1	n/d	n/d	15	n/d	12.8	n/d	11.5	n/d
CRP, mg/L	<1	n/d	n/d	2.2	20.2	<1	<1	n/d	n/d
Sodium, mmol/L	140	137	127	95	131	141	138	138	142

^a^WCC – total white cell count in the blood

^b^n/d – not done
  At the time of presentation serum potassium was 5.2 mmol per litre – upper end of the normal range (Table 1). This notwithstanding, Nandagopal et al. described a case of biochemically confirmed pseudohypoaldosteronism (markedly elevated aldosterone) in a child with E coli UTI and hyponatraemia (plasma sodium of 111 mmol per litre), metabolic acidosis, and potassium of 5.6 mmol per litre [22]; furthermore, Rodriguez-Soriano et al. described 32 children with acute pyelonephritis and normokalaemic pseudohypoaldosteronism [23]. In the case presented here, serum renin and aldosterone were not measured until day four of presentation when the baby had already recovered. Both were raised, however the level of aldosterone was raised only slightly and only when compared to the reference range for infants reported by Nandagopal et al. (Table 1). Notably, when the two urine steroid profiles were compared (Table 4), aldosterone metabolites were only slightly higher in the former sample, indicative of a rather weak aldosterone response to the extreme hyponatraemia and hypovolaemia. This impaired aldosterone response to stimulation has been described elsewhere and is probably due to prematurity [6, 24].

**Table 4:** Urinary steroid profile^a^

Steroid	Day 0, serum	Day 3, serum	Mean (SD), (n = 16)
	Na = 109 mM	Na = 135 mM	
3β-Hydroxy-5-ene steroids (microgram per 100 ml)			
16α–Hydroxy DHA	335	32	303 (291)
16β–Hydroxy DHA	11	6	149 (111)
16-oxoandrostenediol + 15β,16α-Dihydroxy DHA	330	99	263 (254)
5-androstene-3β,16α,17β-triol	116	27	199 (142)
16α,18-Dihydroxy DHA	119	75	426 (341)
16α-Hydroxypregnenolone	385	3	427 (380)
5-Androstene-3β,16α,17β,18-tetrol	48	18	49 (27)
5-Androstene-3β,15β,16α,17β-tetrol	97	41	82 (62)
5-Androstene-3β,15α,16β,17β-tetrol	53	20	40 (23)
5-Pregnene-3β,16α,20α,21-tetrol	18	5	24 (15)
Cortisol metabolites (microgram per 100 ml)			
Tetrahydrocortisone	166	56	108 (85)
α-Cortolone	4	1	32 (53)
β-Cortolone	33	6	20 (17)
1β-Hydroxytetrohydrocortisone	4	2	21 (18)
1β-Hydroxy-β-Cortolone	38	14	25 (22)
6α-Hydroxytetrahydrocortisone	22	16	86 (78)
6α-Hydroxy-α-cortolone	27	10	12 (8)
6α-Hydroxy-β-cortolone	154	67	45 (40)

^a^all measurements were done at Steroid Profiling Laboratory, Department of Biochemistry, King’s College Hospital, London, UK, which also provided the mean and standard deviations (SD) based on 16 controls for reference
  An additional feature that developed following the initial drop of sodium to 127 mmol per litre was metabolic acidosis. As a rule, premature babies at this stage are likely to have developed compensated respiratory acidosis, which was indeed the case with his triplet sisters (not shown). Contrary to that, at the time of the extreme hyponatraemia venous blood gas revealed compensated metabolic acidosis with raised lactate, which normalised within 48 h of treatment (Table 1). Serum chloride was low as is often the case with hyponatraemia and against renal tubular acidosis [25].

**Fig. 6:**
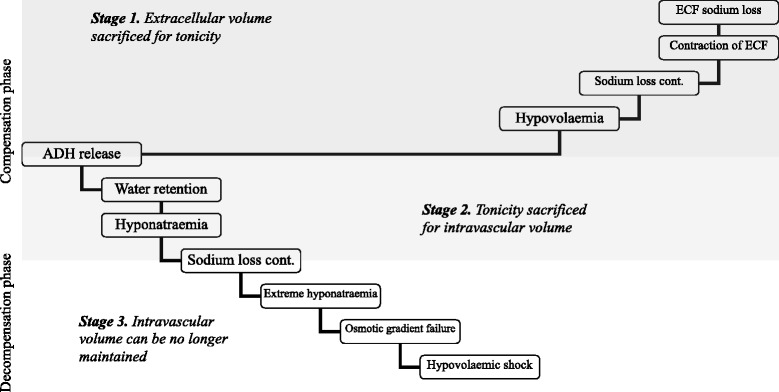
Proposed pathogenesis of hypovolaemic hyponatraemia in this baby

4) *Proposed pathogenesis of hypovolaemic hyponatraemia in this baby*
  Figure 6 charts propositional chain of events, which lead to hyponatraemia and hypovolaemia in this baby as inferred from the available data. The events were triggered by renal sodium loss, probably due to UTI, and augmented by already weak sodium reabsorption characteristic of very premature babies [4]. This led initially to extracellular fluid (ECF) volume contraction, which maintained sodium at low-normal level but resulted in poor weight gain. As the sodium losses continued, circulatory volume began to contract, which led to activation of renin-angiotensin system and release of ADH. Thus, the sodium level was sacrificed in favour of maintaining circulatory volume. This resulted in small weight gain and initial drop of sodium (to 127 mmol per litre). Hyponatraemia was further augmented by the provision of large volume of hypotonic feeds (DBM). Sodium losses and inadequate sodium provision continued and resulted in further exacerbation of hyponatraemia. This led to the failure of the intramedullar osmotic gradient and clinically significant circulatory volume contraction due to impaired renal response to ADH in this environment [26].

### Management of hyponatraemia and hypovolaemia

At the time of presentation, the child’s moribund state, the extreme degree and unknown duration of hyponatraemia prompted concerns regarding acute onset hyponatraemia with impending brain oedema and associated risk of encephalopathy and respiratory arrest [27]. For this reason, in the initial phase of the treatment the aim was to bring plasma sodium into mild hyponatraemia range (120 mmol per litre) relatively quickly [28]. The sodium deficit was estimated at 43.5 mmol ((135 mM – 95 mM) × 1.36 kg × 0.8) and the baby was given 0.9 % sodium chloride infusion intravenously at 16.6 ml per hour aiming at bringing sodium level to 120 mmol per litre within 24 h (increase of around 1 mmol per litre per hour). Unexpectedly, four hours later serum sodium was already 112 mmol per litre (*i.e.* increase of four mmol per litre per hour) – this was later explained by brisk diuresis (see above) - so the infusion rate was halved. In the subsequent 20 h, the rate of plasma sodium increase averaged 0.75 mmol per litre per hour.

Figure 5 summarises sodium provision from the eve of presentation and through the treatment. Any additional sodium supplementation was gradually reduced and stopped on day 11 of treatment: serum sodium remained normal or near normal on all subsequent measurements.

### Clinical progress and long term follow up

Irritability and skin mottling resolved within the first 24–36 h of treatment. On day 3 of treatment, a short episode of twitching of the legs was noted without change of the heart rate or oxygen saturation: this did not reoccur and was neither investigated nor treated.

Weight gain, which was fairly static until presentation despite parenteral nutrition and enteral feeds, improved significantly shortly after treatment (Fig. 2).

Developmentally, at 24 months, as he became more mobile, there appeared to be excess circumduction at the hips along with toe-walking and increased tone in the legs. The hand use was satisfactory and he showed good hand-eye coordination. These features were in keeping with mild diplegic cerebral palsy, typically associated with premature birth [29]. This was further corroborated at the age of four years by findings of periventricular gliosis on magnetic resonance imaging of the brain (not shown).

## Conclusions

Several aspects of this case were instructive and challenging. The duration of hyponatraemia and child’s hydration status are often cited as the key features on which to base management [7]. However, both clinical deterioration and extreme hyponatraemia in this baby were completely unexpected and it was only after several hours that we were able to infer that hyponatraemia was probably chronic (*i.e.* longer than 48 h). The ascertainment of the hydration status was even harder and required combining clinical features with data on weight gain, fluid intake, and biochemical parameters: all this took place at a much later stage.

It has also been noted that cause-specific treatment should be instituted as soon as possible [30]. However, the nature of the condition meant that the diagnostic assessment was going to be protracted with turn-around time for many endocrine tests in excess of two weeks. The sepsis work up and empirical treatment with antibiotics were done as part of a routine management of a sick neonate.

At the time of writing there were no consensus clinical practice guidelines on diagnosis and treatment of hyponatraemia in children but there was one for adults [30]. The authors of the guideline found no specific studies addressing management of hyponatraemia in patients with contracted ECF volume and stated that their recommendations were “based on direct translation of pathophysiology into clinical practice”. They suggested that for the replenishment of water and sodium, isotonic saline was likely to be inferior to balanced crystalloid solutions as it contained very high concentration of chloride, which could impair renal function. They also felt that the need for volume resuscitation in haemodynamically unstable patients overrode concerns for overly rapid correction of sodium and suggested that glucose solutions could be used if “overcorrection was imminent”. Overall prerequisite was management in the environment where close monitoring and ability to monitor sodium frequently was possible. Authors recommended re-lowering the serum sodium concentration if it increased by more than 10 mmol per litre during the first 24 h or by more than 8 mmol per litre in any 24 h thereafter. Authors also recommended obtaining expert advice on the use of electrolyte-free solutions (such as glucose infusions) and on the use of intravenous desmopressin.

Clearly, whilst the baby did not come to harm, the sodium correction rate was too fast compared to the correction rate suggested for adults with hypovolaemic hyponatraemia and had the authors been aware of the recommendations, it is likely that the sodium correction rate would have been aimed at 10 – 8 mmol per litre per day. In addition, we were not aware of the need to anticipate brisk diuresis after the commencement of the treatment and hence were “caught” by the unexpectedly fast increase of the sodium level in the blood. Again, it is likely that we would have contemplated using 5 % glucose infusion instead of 0.9 % sodium chloride as it has been suggested for adults with this condition [30]. We state these, fully aware that direct translation of adult-orientated guidance to premature babies, albeit with a similar condition, is unlikely to be prudent.

As reported by Shaffer and colleagues, late hyponatraemia and hypovolaemia in premature babies is not uncommon: 6 out of 18 infants born at 32 weeks gestational age in their series [6]. This finding indicates that in premature babies endocrine reactions often do not normalize sodium and water balance but lead to sodium chloride losses; this could be due to weak response of the premature adrenal glands [1]. This, as well as other peculiarities such as weak reabsorption in proximal and distal tubules [1, 4], high rate of urinary sodium excretion in sodium-restricted infants [31], weak concentrating ability of the kidneys [32], call for evidence-based hyponatraemia guidance applied to children and premature babies specifically. We hope the case reported here would contribute to that goal.

## Consent

Written informed consent was obtained from the parents of this baby for publication of this case report and any accompanying images. A copy of the written consent is available for review by the Editor of this journal.

## Abbreviations

DBM: Donor breast milk
CPAP: Continuous positive airway pressure
ISE: Ion specific electrodes
ADH: Antidiuretic hormone
EBM: Expressed breast milk
FENa: Fractional excretion of sodium
CAH: Congenital adrenal hyperplasia
HHS: Hyponatraemic hypertensive syndrome
UTI: Urinary tract infection
CRP: C-reactive protein
WCC: White cell count
ECF: Extracellular fluid
